# Adolescents’ perceptions of active school transport in northern Sweden

**DOI:** 10.1016/j.heliyon.2023.e20779

**Published:** 2023-10-07

**Authors:** Nuru Jingili, Solomon Sunday Oyelere, Simon Malmström Berghem, Robert Brännström, Teemu H. Laine, Oluwafemi Samson Balogun

**Affiliations:** a Department of Computer Science, Electrical and Space Engineering, Luleå University of Technology, 931 87, Luleå, Sweden; b Department of Digital Media, Ajou University, Suwon, 16499, South Korea; c School of Computing, University of Eastern Finland, Kuopio, Finland

**Keywords:** Active school transport, Adolescent, Physical activity, Walking, Cycling, Riding a non-motorised scooter

## Abstract

Active school transport (AST) refers to using active means of transport such as walking, cycling, or riding a non-motorised scooter to school. It can help improve adolescents’ physical activity levels and create a more sustainable environment. The study involved 70 adolescents (45 boys and 25 girls) aged 13 to 14 from one school in Skellefteå, in Northern Sweden. In an online questionnaire, they were asked about their perceptions of cycling, walking, and riding a non-motorised scooter to school. This study used descriptive statistics, multiple regression analysis, and hypothesis testing with ANOVA to analyse the collected data and compare the perceptions of different types of transport on safety, environmental, and personal factors among adolescents in Northern Sweden. According to the results, more adolescents walked to school than cycled, and significantly few rode a non-motorised scooter to school. Most adolescents believe walking or cycling to school is a great way to exercise. Furthermore, the study also revealed that many adolescents avoid using AST due to the time it takes. Although the study showed that adolescents felt sufficient support for using AST from schools and parents, the number of adolescents using motorised transport is higher during winter than in summer. Additionally, most of them were more confident about cycling and walking to school than riding a non-motorised scooter and thought using AST was nice. Finally, most adolescents perceived having complete control over their transport options when going to school. The research indicates that it is crucial to implement interventions that inspire children to be interested and excited about using AST. These strategies should include fostering an AST culture that is fun and positive, as well as creating environments that are safe and supportive. The research results will guide the creation of a persuasive game that can motivate adolescents to use AST and measure its effectiveness.

## 1 Introduction

Commuting actively to school (referred to henceforth as active school transport, AST) by non-motorised means such as walking, cycling or scooter can increase the levels of physical activity [[1], [2], [3]] create a greener and sustainable environment [[4], [5], [6]], and improve the cardiovascular and mental health of children [7,8]. Besides, non-motorised transport has been recommended as a vital means to minimize greenhouse gas emissions associated with transport [9], and the effect of climate change can be alleviated through the adoption of game-based interventions for AST [10], practices and behavioural aspects. The popularity of active commuting differs significantly between countries. A multi-country study indicated that Finland and Colombia, 79.4 % and 73.8 %, respectively, have the highest rates of AST, while India and the United States, with 5.2 % and 10.8 %, respectively, have the lowest rates of AST [3]. A similar study of 9–11-year-old children from 12 countries recorded that roughly 42 % of children travelled to school by AST mode (walking, bicycle, roller-blade, skateboarding, or non-motorised scooter), 57 % by non-AST mode (car, motorcycle, bus, train, tram, underground, boat, or moped), and 1 % travelled by some other mode [11]. In Sweden, active commuting decreased with age, and the use of public transport increased among children [12].

Previous studies have shown several correlates and indices that are considered in determining the AST among children. These include sociodemographic characteristics, personal and family factors, school conditions, and environmental factors [13,14]. Previous studies have investigated the impact of walking and cycling [15]. However, only a handful of studies have focused on new modes of AST, such as non-motorised scooters and skateboards [16]. In this study, the three modes of transportation under investigation are walking, cycling, and using non-motorised scooters. These methods were specifically chosen because they are considered active modes of transportation, meaning they require physical effort and contribute to a more active lifestyle. The focus on these three modes of travel is due to their popularity, feasibility, and relevance in the context of school commuting. Each AST mode requires different behaviours by children, which correspond to likely different correlates [8,11,16]. Mostly, children's AST has been measured based on self-reports from children or their parents, and better results can be obtained among older children [[17], [18], [19]]. An essential barrier to AST and a predictor of the mode of transport among adolescents is the distance [2,20]. According to Nelson et al. (2008), the optimum distance for AST among 15-17 year-olds is 2.5 miles (4 km); however, the optimum distance is likely to be lower for younger children.

In northern Sweden, between 50 % and 76 % of schoolchildren have been reported using AST depending on their age [19] and the time of year [1,21]. The decision to either walk, cycle or use a non-motorised scooter to school is largely dependent on socio-demographic, socio-economic, and built environment characteristics [19]. Besides, parental attitudes have positively improved towards their children using AST [1]. Encouraging and promoting the use of AST among children and adolescents is paramount for fostering healthy habits, reducing environmental impact, and nurturing a sense of independence and responsibility from an early age. This study delves into the perceptions and attitudes of adolescents towards different modes of AST, shedding light on the factors that influence their choices and the barriers they face. By understanding these dynamics, the study contributes to the development of effective strategies to motivate and promote the adoption of AST practices among children. A previous study found an association between lower socioeconomic position and increased AST in urban areas in Sweden. However, there is a lack of study about how AST varies based on other indices and modes of transport. Therefore, this study aimed to investigate and compare the perceptions of three modes of AST, walking, cycling, and non-motorised scooters, in consideration of additional personal, environmental, and safety indicators among children from northern Sweden. This study addresses the following research questions:

1. How do adolescents' perceptions of safety, environmental impact, and personal factors vary between different modes of transport to school in Northern Sweden?
2. Is there a relationship between distance travelled to school and the choice of the mode of transport?
3. Does a statistically significant relationship exist between walking and cycling and riding non-motorised scooter to school?

## 2 Methods

### 2.1 Procedure and participants

In a school located in the northern part of Sweden, specifically in the Skellefteå municipality, a group of children aged 13 to 14 were chosen to participate in this research. The children who participated in this research were all in grade eight. The primary language these students speak is Swedish, but all students have a fair understanding of English. Both male and female students were included in this study. The study involved both boys and girls from this school. Skellefteå, the city where this study took place, has around 73,984 people. It experiences a subarctic climate, meaning its summers are relatively mild, while its winters are cold and covered in snow. The participants anonymously answered an online questionnaire about AST on Google Forms under the supervision of both researchers and teachers in two sessions. The first session there were 70 participants, and consisted of five teachers and four researchers, two of whom were native Swedish speakers. In the second session, there were only 65 adolescent children from the same class, and their gender was not recorded. It is also unknown if there were any new children who were not present during the first session. The second session included five teachers and five researchers, three of whom were native Swedish speakers. The participants were divided into four classes, with one teacher and researcher present in each class, while one teacher visited all the classes. The data collected was then translated into English by the same researcher who translated the questionnaire for reporting purposes. The questionnaire was developed in English and translated into Swedish by one of the researchers, who was a native Swedish speaker with fluency in English. The researchers guided the participants through the process, ensuring their comprehension of the questions, and observed verbal and physical reactions. The questionnaire was completed by the participants in 5–10 min.

### 2.2 Ethical consideration

Doing research with minors requires special attention and following proper ethical guidelines. Therefore, this study was conducted according to the Code of Ethics of the World Medical Association (Declaration of Helsinki), even though it did not collect personal health data. For this event, we only collected data after receiving informed consent from the children's guardians and teachers. No personal information was collected during the event, and on their demographic information, we only recorded the genders and ages of the participants. The data collected on Google Forms were anonymous, and only the researchers had access to them.

### 2.3 Questionnaire

The participants were asked to complete a questionnaire that included their sociodemographic characteristics, such as their age, gender, and home resources. They were also asked about their travel habits, attitudes towards walking, cycling, and riding non-motorised to school, perceived or subjective norms, personal incentives, personal barriers, environmental factors, and safety perceptions towards AST. The following sections present the components of the questionnaire in detail.

#### 2.3.1 Sociodemographic characteristics

We collected information about the participants’ sociodemographic characteristics, including age, gender, and home resources [13]. In the home resources, we asked the participants to provide the number of bicycles, vehicles, motorised scooters, non-motorised scooters, and waterproof raincoats at their homes [13]. In addition, we asked them to provide an estimated distance to the school (km).

#### 2.3.2 School transportation habits

The questionnaire asked the participants about their usual way of travelling to school and back home so as to determine whether they used active or passive modes of transport to school [13,21]. The responses were categorised into five: never, rarely, sometimes, most of the time, and all of the time. Participants were able to rate how often they used each mode of the transport mentioned. We used multi-modal and dominant modes of transport to classify the adolescents into active transport categories if they used it most or all of the time. The AST options included walking, cycling, and riding a non-motorised scooter to school. The questionnaire also asked the participants about the frequency of using AST. For this question, the expected responses were never, almost never, sometimes, or almost every day. They were also asked to provide an estimated time it would take them to reach school if they walked or cycled or rode a non-motorised scooter to school. In this case, the responses included 1–5 min, 6–10 min, 11–20 min, 21–30 min, 31+ min, and I don't know.

#### 2.3.3 Attitudes towards walking, cycling, and riding non-motorised to school

The questionnaire also asked the participants about their attitudes toward cycling, walking, and riding a non-motorised scooter to school. In this, the participants were asked to rate statements on whether regularly walking, cycling and riding a non-motorised scooter to school would be dull or interesting, unpleasant or pleasant, boring or stimulating, unhealthy or healthy, bad or good, and useless or useful [13,22]. A 6-point Likert scale with strongly disagree, disagree, somewhat disagree, somewhat agree, agree, and strongly agree was used to measure the responses for each mode of transport.

#### 2.3.4 Perceived or subjective norms

The questionnaire included statements for assessing subjective norms towards walking and cycling to school. The following statements were presented to the participants: My parents or guardians think I should walk, cycle or ride a non-motorised scooter to school, My friends think I should cycle or ride a non-motorised scooter to school, One or both of my parents/guardians walk, cycle or ride a non-motorised scooter [13,23]. We also included statements like It is not considered cool to walk, cycle or ride a non-motorised scooter to school, No other students walk, cycle or ride a non-motorised scooter to school, My school encourages me to walk, cycle or ride a non-motorised scooter to school, I am confident I could walk, cycle or ride a non-motorised scooter to school, I have complete control over whether or not I walk, cycle or ride a non-motorised scooter to school, and I intend to walk, cycle or ride a non-motorised scooter to school [13,22]. A 7-point Likert scale with strongly disagree, disagree, somewhat disagree, neutral, somewhat agree, agree, and strongly agree was used to measure the responses for each mode of transport.

#### 2.3.5 Personal incentives and barriers

The questionnaire included statements for measuring personal incentives like walking, riding a bicycle and a non-motorised scooter to school is a great way to get some exercise [6,13]. On the other hand, the measure of personal barriers included statements like walking and cycling and riding a non-motorised scooter to school takes too much time [6,13], involves too much planning ahead to walk to school, and I get too hot and sweaty [13]. A 7-point Likert scale strongly disagree, disagree, somewhat disagree, neutral, somewhat agree, agree, and strongly agree was used to measure the responses for each mode of transport.

#### 2.3.6 Environmental factors and safety perceptions

The study included questions for assessing environmental factors, particularly the distance, availability of footpaths, cycle paths and non-motorised scooter paths and weather effects [6,23]. In measuring safety perceptions, we included statements that assessed the participants’ perceptions of safety in walking, cycling and riding a non-motorised scooter [13]. A 7-point Likert scale strongly disagree, disagree, somewhat disagree, neutral, somewhat agree, agree, and strongly agree was used to measure the responses for each mode of transport.

### 2.4 Data analysis

We used descriptive statistics to analyse the demographic characteristics. We also used multiple regression analysis to determine the relationship between adolescents’ perceptions of active school transport (dependent variable) and the distance adolescents travelled to school, number of people who cycle, walk, use public transport, car, bicycles, and non-motorised scooter to school (independent variables) during the summer and winter. In addition, Pearson correlation analysis was employed to ascertain the link between the mode of transportation and attitudes. Multiple regression analysis allowed us to analyse the effect of independent variables on the dependent variable [24]. Furthermore, we used a one-way ANOVA for continuous variables. In reporting descriptive data for continuous variables, we used mean ± standard deviation and used frequencies for categorical variables. The data analysis was performed using the SPSS Statistical Package, version 28, and statistical significance was set at a p-value of <0.05.

## 3 Results

### 3.1 Sociodemographic

Table 1 summarises the sociodemographic characteristics of the participants. During the first session, 70 adolescent children aged 13–14 with a mean of 13.91 ± 0.282 participated, with 45 boys (64.3 %) and 25 girls (35.7 %). Most adolescents live within 5 km of school (64.3 %), and only about 35.7 % live further away. Around 90 % of adolescents had multiple bicycles at home, while more than 90 % of adolescents had at least one vehicle in their homes. Furthermore, about half of them had one or more non-motorised scooters at home, while only 21.4 % had motorised scooters. Over 90 % of adolescents had multiple waterproof raincoats.

**Table 1:** Sociodemographic characteristics.

Study sample n = 70	
Age(years) [ Mean ± SD]	13.91 ± 0.282
**Gender [n (%)]**	
Boy	45 (64.3 %)
Girl	25 (35.7 %)
**Distance to school (km) [n (%)]**	
Between 0 and 1 km	26 (37.1 %)
More than 1–2 km	13 (18.6 %)
More than 2–5 km	6 (8.6 %)
More than 5 km	25 (35.7 %)
**Number of bicycles at home [n (%)]**	
None	1 (1.4 %)
One	3 (4.3 %)
Two or more	63 (90 %)
Missing information	3 (4.3 %)
**Number of vehicles at home [n (%)]**	
None	1 (1.4 %)
One	17 (24.3 %)
Two or more	50 (71.5 %)
Missing information	2 (2.9 %)
**Number of non-motorised scooters at home [n (%)]**	
None	33 (47.1 %)
One	15 (21.4 %)
Two or more	20 (28.6 %)
Missing information	2 (2.9 %)
**Number of motorised scooters at home [n (%)]**	
None	55 (78.6 %)
One	11 (15.7 %)
Two or more	4 (5.7 %)
**Number of waterproof raincoats at home [n (%)]**	
None	2 (2.9 %)
One	2 (2.9 %)
Two or more	64 (91.52 %)
Missing information	2 (2.9 %)

### 3.2 Travel to school habits

The questionnaire results reveal that most participants usually travel to school using public transport, on foot or by bicycle during summer and winter (Table 2). Moreover, travelling to school-by-school bus or car is more common in winter than in the summertime. There is not much difference in cycling habits between summer and winter among adolescents (cycling in summer: 28.6 %, cycling in winter: 30.0 %). On the other hand, more adolescents walked to school in winter (54.2 %) than in summer (37.1 %). The use of non-motorised scooters is rare among adolescents regardless of the weather conditions. Only about 1.4 % used non-motorised scooters to school (Table 2). During summer, about 37.1 % of adolescents used active transport all of the time, while 12.9 % used motorised transport all of the time and 50 % used combined motorised and active transport (Table 2). In winter, about 31.4 % of adolescents used active transport all of the time, while 27.1 % used motorised transport all of the time and 41.5 % used combined motorised and active transport (Table 2).

**Table 2:** Travel to school habits.

Study sample (n = 70)	
Usually travel to school during summer [n (%)]	
By car	11 (15.6 %)
By school bus	12 (17.1 %)
By public transport	23 (32.9 %)
On foot	26 (37.1 %)
By bike	20 (28.6 %)
By motorised scooter	3 (4.3 %)
By non-motorised scooter	1 (1.4 %)
By skateboard	3 (4.3 %)
**Usually travel to school during winter [n (%)]**	
By car	30 (42.9 %)
By school bus	18 (25.7 %)
By public transport	27 (38.6 %)
On foot	39 (54.2 %)
By bike	21 (30 %)
By motorised scooter	1 (1.4 %)
By non-motorised scooter	1 (1.4 %)
By skateboard	1 (1.4 %)
**Active and motorised transport to school during summer [n (%)]**	
Active transport	26 (37.1)
Motorised transport	9 (12.9 %)
Combination of active and motorised transport	35 (50 %)
**Active and motorised transport to school during winter [n (%)]**	
Active transport	22 (31.4 %)
Motorised transport	19 (27.1 %)
Combination of active and motorised transport	29 (41.5 %)

The questionnaire results showed that 53.1 % of the adolescents could walk to school while 60.1 % could cycle to school, and 47.2 % could ride a non-motorised scooter to school in about 20 min (Table 3). Table 3 shows that 25.7 % of the respondents did not know how long it would take to ride a non-motorised scooter to school this could be because they have not ridden a non-motorised scooter before.

**Table 3:** Estimated time to travel to school.

Study sample (n = 70)			
Estimated time to travel to school<	Walking n (%)	Cycling n (%)	Riding non-motorised scooter n (%)<
1–5 min	13(18.8)	20 (28.6)	18(25.7)
6–10 min	7 (10.0)	15 (21.4)	9(12.9)
11–20 min	17 (24.3)	7 (10.1)	6(8.6)
21–30 min	4 (5.7)	18 (25.7)	5(7.1)
31+ min	24 (34.3)	6 (8.6)	14(20.0)
I don't know	5 (7.1)	4 (5.7)	18(25.7)

Table 4 shows the travel habits of the previous two weeks. The responses show that only 35.8 % of adolescents walked to school almost every day, while 51.4 % almost never walked. Similarly, 58.6 adolescents never cycled to school compared to 22.8 % who cycled almost every day. During the previous two weeks that the data collection was carried out, no one used a non-motorised scooter to school.

**Table 4:** Transport to school in the previous two weeks.

Study sample n = 70			
Transport to school in the previous two weeks<	Walking to School n (%)	Cycling to School n (%)	Riding non-motorised scooter to school n (%)<
Never	31(44.3)	41 (58.6)	70(100)
Almost never	5 (7.1)	8 (11.4)	
Sometimes	9 (12.9)	5 (7.1)	
Almost every day	9 (12.9)	11(15.7)	
Every day	16 (22.9)	5 (7.1)	

The results shows that 36 % of the variability in the Adolescents’ perceptions of active school transport is explained by the distance adolescents travelled to school, number of adolescents who cycle, walk, use public transport, bus, car, and non-motorised scooter (Table 5). The regression model significantly fits the data with the p-value being 0.001 (Table 6).

**Table 5:** Model summary.

Model	R	R square	Adjusted R squared
1	0.596	0.356	0.271

**Table 6:** Analysis of Variance for the fitted Regression model.

Model	Sum Squares	df	Mean Square	F	p
Regression	10287.554	8	1285.944	4.211	0.000
Residual	18628.289	61	305.382		
Total	28915.843	69			

There is a significant relationship between the Adolescents’ perceptions of active school transport and the number of adolescents who walk and cycle to school during summer and winter while there was no significant relationship between the distance to school and number of adolescents using use public transport, bus, car and non-motorised scooter to school during summer and winter and distance to school (Table 7).

**Table 7:** Test for equality of means.

	Unstandardized coefficient B	Std error	Standardized coefficient Beta	t	p
Constant	69.331	16.353		4.240	0.000
Walk	2.663	1.130	0.390	2.356	0.022
Bicycles	3.988	1.216	0.449	3.280	0.002
Non-motorised scooter	−0.098 0.923	4.358 3.844	−0.005 0.049	−0.022 0.240	0.982 0.811
Bus	−0.079	0.991	−0.010	−0.080	0.937
Car	−1.048	1.062	−0.107	−0.986	0.328
Public transport	0.110	1.047	0.017	0.105	0.916
Distance to School	0.624	1.007	0.105	0.620	0.538

### 3.3 Adolescents’ perceptions of walking, cycling and riding non-motorised scooters to school

The findings, as detailed in Table 8, highlight several interesting perceptions. A substantial percentage of participants found the idea of walking (60 %), cycling (72.9 %), and using non-motorised scooters (78.6 %) to commute to school interesting. Similarly, a notable number of participants expressed that walking (65.7 %) and cycling (87.1 %) to school were pleasant. However, the pleasantness perception was less prominent for riding non-motorised scooters to school, with less than half of the participants indicating such sentiments. Conversely, a significant proportion of participants reported finding walking (52.9 %) and cycling (75.7 %) to school somewhat boring. Interestingly, a mere 28.6 % of participants viewed riding non-motorised scooters to school as a dull experience. When considering the aspect of health, an overwhelming majority of participants (95.7 %) believed that walking to school promotes well-being. Similarly, an even higher percentage (98.6 %) attributed positive health effects to cycling. However, this perception was less pronounced for using non-motorised scooters (64.3 %). Moreover, concerning the perceived benefit of these transportation modes, a substantial majority found walking (82.9 %) and cycling (95.7 %) to school good. On the other hand, less than half of the participants (47.1 %) held the same view regarding using non-motorised scooters for their school commute. Most participants across the board did not perceive walking, cycling, or using non-motorised scooters as particularly useful for their school travel, as outlined in Table 8.

**Table 8:** Perceived norms towards walking, cycling and riding non-motorised to school.

Total sample (n = 70)	Walking				Cycling				Riding non-motorised scooter				
	Mean ± SD	Disagree n(%)	Neutral n(%)	Agree n(%)	Mean ± SD	Disagree n(%)	Neutral n(%)	Agree n(%)	Mean ± SD	Disagree n(%)	Neutral n(%)	Agree n(%)	P-value
**Attitudes**													
For me, regularly walking/cycling/Riding a non-motorised scooter to school would be interesting^a^	3.80 ± 1.48	28(40)	0	42(60)	2.81 ± 1.34	19(27.1)	0	51(72.9)	4.36 ± 1.24	15(21.4)	0	55(78.6)	0.001
For me, regularly walking/cycling/Riding a non-motorised scooter to school would be pleasant^a^	3.66 ± 0.48	24(34.3)	0	46(65.7)	3.87 ± 0.34	9(12.9)	0	61(87.1)	3.49 ± 0.50	36(51.4)	0	34(48.6)	0.001
For me, regularly walking/cycling/Riding a non-motorised scooter to school would be boring^a^	5.53 ± 0.50	33(47.1)	0	37(52.9)	5.76 ± 0.43	17 (24.39	0	53(75.7)	5.29 ± 0.46	50(71.4)	0	20(28.6)	0.001
For me, regularly walking/cycling/Riding a non-motorised scooter to school would be healthy^a^	1.96 ± 0.20	3(4.3)	0	67(95.7)	1.99 ± 0.12	1(1.4)	0	69(98.6)	1.64 ± 0.48	25(35.7)	0	45(64.3)	0.001
For me, regularly walking/cycling/Riding a non-motorised scooter to school would be good^a^	3.83 ± 0.38	12(17.1)	0	58(82.9)	3.96 ± 0.20	3(4.3)	0	67(95.7)	3.47 ± 0.50	37(52.9)	0	33(47.1)	0.001
For me, regularly walking/cycling/Riding a non-motorised scooter to school would be useful^a^	2.3 ± 0.90	63(90)	0	7(10)	2.17 ± 0.70	66(94.3)	0	4(5.79)	3.16 ± 1.47	43(61.4)	0	27(38.6)	0.001
**Subjective/perceived norms**													
My parents or guardians think I should^b^	4.91 ± 2.12	13(18,6)	20(28.6)	37(52.8)	5.51 ± 1.65	5(7.2)	20(28.6)	45(64.3)	2.96 ± 1.81	34(48.6)	28(40)	8(11.4)	0.001
My friends think I think I should walk/ride a bicycle/non-motorised scooter to school^b^	4.40 ± 1.92	13(18,6)	33(47.1)	24(34.3)	5.03 ± 1.65	5(7.2)	32(45.7)	33(47.1)	2.94 ± 1.70	32(45.8)	33(47.1)	5(7,2)	0.001
One or both of my parents or guardians walk/ride a bi- cycle/non-motorised scooter frequently^b^	4.46 ± 2.26	20(28.6)	15(21.4)	35(50)	4.67 ± 2.01	14(20)	21(30.0)	35(50.0)	1.76 ± 1.52	57(81.2)	8(11.4)	5(7.1)	0.001
It is not considered cool to walk/ride a bicycle/non-motorised scooter to school^b^	2.56 ± 1.81	41(58.6)	23(32.9)	6(8.6)	2.60 ± 1.81	40(57.1)	24(34.3)	6(8.6)	2.90 ± 1.87	39(55.7)	22(31.4)	9(12.8)	0.485
No other students walk/ride. a bicycle/non-motorised to school^b^<	2.30 ± 1.62	46(65.7)	20(28.6)	4(5.7)	2.29 ± 1.70	49(70.0)	16(22.9)	5(7.2)	4.16 ± 2.40	24(34.3)	21(30.0)	25(35.7)	0.001
My school encourages me to walk/ride a bicycle/non-motorised scooter to school^b^	4.41 ± 1.83	11(15.6)	33(47.1)	26(37.2)	4.53 ± 1.75	10(14.3)	31(44.3)	29(41.4)	3.30 ± 1.72	24(34.2)	39(55.7)	7(10.0)	0.001
**Perceived behavioural control**													
I am confident I could walk/ride a bicycle/non- motorised scooter to school^b^	4.61 ± 2.01	18(25.7)	7(10.0)	45(64.3)	6.03 ± 1.73	6(8.5)	6(8.6)	58(82.8)	4.51 ± 2.40	21(30.0)	13(18.6)	36(51.4)	0.001
I have complete control over whether or not I walk/ride a bicycle/non-motorised to school^b^	5.61 ± 1.89	7(10)	14(20.0)	49(70.0)	5.84 ± 1.59	0	NA	0.0	4.47 ± 2.39	21(30.0)	16(22.9)	9(47.1)	0.001
**Behavioural intentions**													
I want to regularly walk/ride a bicycle/non-motorised scooter to school^b^<	3.91 ± 2.32	28(40)	14(20.0)	28(40)	4.87 ± 2.08	14(20)	18(25.7)	38(54.3)	1.81 ± 1.55	49(84.2)	6(8.6)	5(7.2)	0.001
I intend to walk/ride a bicycle/non-motorised scooter to school frequently^b^	4.39 ± 2.33	22(31.4)	15(21.4)	33(47.1)	5.07 ± 2.01	11(15.7)	19(27.1)	40(57.2)	1.94 ± 1.48	54(77.1)	13(18.6)	3(4.3)	0.001

a Data collected using a 6-point Likert scale (1 = strongly disagree to 6 = strongly agree). Data recorded as 1,2,3 = disagree, and 4,5,6 = agree to create categorical variables.

b Data collected using a 7-point Likert scale (1 = strongly disagree to 7 = strongly agree). Data recorded as 1,2,3 = disagree, 4 = neutral, and 5,6,7 = agree to create categorical variables.

Generally, participants perceived to have received considerable parental, peer and school support for walking and cycling than for riding a non-motorised scooter to school (Table 8). Compared to riding a non-motorised scooter to school, adolescents were more optimistic about walking and cycling. They also believed walking, cycling, and riding a non-motorised scooter to school were cool. However, 8.6 % of them also said it was not cool to cycle and walk to school, and 12.8 % said it was not cool to ride a non-motorised scooter to school. The percentage of adolescents who said they were confident about their ability to cycle (82.8 %) and walk (64.3 %) to school was higher than those riding a non-motorised scooter (51.4 %). Similarly, many adolescents said they have complete control over whether to walk or cycle to school than ride non-motorised scooters to school (Table 8). Adolescents' intentions were lower for riding a non-motorised scooter (7.2 % and 4.3 %) than for walking (40 % and 47.1 %) and cycling (54.3 % and 57.2 %) to school. The result from the ANOVA showed perceived norms are statistically significant except for walking, cycling, and riding a non-motorised scooter to school is not considered cool. Appendix 1 shows in detail the pairs that are significant or not for Table 8.

Adolescents perceive cycling (52 %) and walking (50 %) to school as a great way of exercising as compared to riding a non-motorised scooter (20 %) (Table 9). Compared to cycling and riding a non-motorised scooter, walking to school has been said to take too much time (Table 9). One personal barrier that is common between walking, cycling, and riding a non-motorised scooter is that some of the participants could not be bothered to use AST (Table 9). On environmental factors, the weather being too cold and wet was seen as a barrier to walking, cycling, and riding a non-motorised scooter to school. On a positive note, about three-quarters of the participants felt it is safe to use AST and felt that their parents thought that it was safe for them to use AST (Table 9). The analysis of variance revealed that not all of the variables were statistically significant. Appendix 2 shows in detail the pairs that are significant or not for Table 9. We have performed a pearson correlation in Table 10, and result shows there is a weak negative correlation between attitudes and mode of transportation. Similarly, it shows there is a significant relationship between the attitudes and mode of transportation with (p = 0.048) < 0.05 (Table).

**Table 9:** Personal Incentives, personal barriers, environmental factors and safety perceptions of walking and cycling to school.

Total sample (N = 65)													
	Walking			Cycling				Riding non-motorised scooter					
Personal incentives	mean ± SD	Disagree n(%)<	Neutral	Agree n(%)<	mean ± SD	Disagree n(%)<	Neutral	Agree n(%)<	mean ± SD	Disagree n(%)<	Neutral	Agree n(%)<	P-value<
Walking/cycling/Riding a non-motorised scooter to school is a great way to get some exercise	5.63 ± 1.36	3(4.6)	12(18.5)	50(76.9)	5.69 ± 1.54	5(7.7)	8(12.3)	52(80)	3.74 ± 1.99	23(35.4)	22(33.8)	20(30.8)	0.001
**Personal barriers**													
Walking/cycling/Riding a non-motorised scooter to school takes too much time	4.43 ± 2.55	27(41.5)	0	38(58.5)	2.51 ± 2.01	52(80.0)	0	13(20)	3.54 ± 2.55	37(56.9)	0	28(43.1)	0.001
It involves too much planning ahead to walk/cycle/ride a non-motorised scooter to school	3.17 ± 2.35	43(66.2)	0	22(33.8)	2.43 ± 1.89	53(81.5)	0	12(18.5)	2.94 ± 2.20	46(70.8)	0	19(29.2)	0.140
I get too hot and sweaty walking/cycling/Riding a non- motorised scooter to school	2.68 ± 2.04	49(75.4)	0	16(24.6)	3.11 ± 2.23	43(66.2)	0	22(33.8)	2.68 ± 2.04	50(76.9)	0	15(23.1)	0.405
I have too much stuff to carry to walk/cycle/ride a non- motorised scooter to school	3.12 ± 2.25	43(66.2)	0	22(33.8)	2.65 ± 2.01	50(76.9)	0	15(23.1)	3.28 ± 2.37	41(63.1)	0	24(36.9)	0.243
It is not convenient for me to walk/cycle/ride a non- motorised scooter to school because of my after-school schedule	2.63 ± 2.10	50(76.9)	0	15(23.1)	2.31 ± 1.91	54(83.1)	0	11(16.9)	2.49 ± 1.98	51(78.5)	0	14(21.5)	0.654
I often feel too tired to walk/cycle/ride a non- motorised scooter to school to school	3.17 ± 2.38	42(64.6)	0	12(35.4)	2.72 ± 2.08	49(75.4)	0	16(24.6)	2.98 ± 2.34	45(69.2)	0	20(30.8)	0.534
I often cannot be bothered to walk/cycle/ride a non- motorised scooter to school	4.12 ± 2.40	23(44.6)	0	36(55.4)	3.34 ± 2.32	41(63.1)	0	24(36.9)	4.66 ± 2.48	24(36.9)	0	41(63.1)	0.008
**Environmental factors**													
It is too far to walk/cycle/ride a non-motorised scooter to school	3.83 ± 2.57	34(52.3)	0	31(47.7)	2.62 ± 2.03	50(76.9)	0	15(23.1)	3.28 ± 2.40	41(63.1)	0	24(36.9)	0.014
There are no footpath/cycle paths to walk/cycle/ride a non- motorised scooter along the way	1.77 ± 1.37	60(92.3)	0	5(7.7)	1.82 ± 1.47	59(90.8)	0	6(9.2)	2.05 ± 1.75	57(87.7)	0	8(12.3)	0.550
wet to walk/cycle/ride a non- motorised scooter	3.82 ± 2.46	34(52.3)	0	31(47.7)	4.11 ± 2.41	30(46.2)	0	35(43.8)	4.34 ± 2.44	28(43.1)	0	37(56.9)	0.474
**Safety perceptions**													
It is unsafe to walk/cycle/ride a non-motorised scooter to school	2.14 ± 1.85	55(84.6)	0	10(15.4)	1.85 ± 1.52	59(90.8)	0	5(9,2)	2.40 ± 2.02	52(80)	0	13(20)	0.222
My parents think it is not safe to walk/cycle/ride a non- motorised scooter to school	1.94 ± 1.55	58(89.2)	0	7(10.8)	1.86 ± 1.46	58(90.8)	0	6(9.2)	2.35 ± 1.92	53(81.5)	0	12(18.5)	0.195

Data collected using a 7-point Likert scale (1 = strongly disagree to 7 = strongly agree). Data was recorded as 1,2,3 = disagree, 4 = neutral, and 5,6,7 = agree to create categorical variables.

**Table 10:** Pearson correlation.

Variables	Pearson correlation	P
Attitude and mode of transportation	−0.237	0.048

## 4 Discussion

### 4.1 Adolescents travel habits

Our study examined the effects of distance to school on the motivation toward AST. Our result was consistent with previous studies that distance significantly predicts children's motivation towards the use AST. There is less motivation towards AST when the distance to school increases and more motivation towards AST when the distance to school decreases [2,6,20]. The built environment and community in Northern Sweden, support physical activity and the promotion of AST. However, in many countries prevalence of using AST is also lower during the winter as the weather condition is seen as a barrier [1,25]. The authors noted that adolescents' AST could increase if they live a few kilometres from school.

Policymakers can use these findings to plan and develop communities with well-designed infrastructure, such as sidewalks, bike lanes, and safe crossings, to encourage children to walk, cycle or ride non-motorised scooters to school. This could reduce the perception of the distance between home and school, making AST more feasible and more attractive to students. Moreover, school authorities can create gamified programs that incentivise students to use AST, such as providing rewards for regular AST users or organising group walks, cycling or riding non-motorised scooter events. Furthermore, parents can play a vital role in promoting AST by encouraging their children to walk, cycle or ride a non-motorised scooter to school and ensuring their safety. Parents can also advocate for safer and more accessible routes to school in their communities.

### 4.2 Adolescents perceptions

The relationship between attitudes and the mode of transportation in AST is a crucial factor influencing whether children and adolescents opt for walking, cycling, or using non-motorised scooters to travel to school. Numerous active transport research studies have researched how the A influence AST behaviour [13,23,26]. The theory of planned behaviour posits that attitudes towards a behaviour, combined with subjective norms and perceived behavioural control, influence that behaviour [27]. In the context of AST, this implies that attitudes towards walking, cycling, and non-motorised scooter usage could shape the decision-making process regarding transportation modes for school transport. Parkany et al. (2004) proposed a circular process involving a causal feedback loop. This model suggests that travel experiences generate positive and negative emotions tied to the chosen mode of transport, thereby shaping perceptions and attitudes towards that mode of transport [28]. These evolving attitudes ultimately influence future travel behaviour choices. Our findings reveal that positive attitudes, encompassing a sense of interest, pleasure, and perception of health, are strongly associated with adopting active modes of transportation, such as walking, cycling, and using non-motorised scooters. These attitudes are vital influencers in the decision-making process, emphasising the importance of cultivating an environment where these modes of transport are perceived as engaging, enjoyable and healthy options for school transport. Conversely, the study highlights that negative attitudes, marked by feelings of boredom, can act as deterrents, particularly for walking and cycling. Understanding these sentiments and their underlying causes is vital for addressing barriers to adopting active transportation modes. By fostering positive attitudes through educational campaigns, awareness programs, and community initiatives, stakeholders can pave the way for healthier, eco-friendly, and more enjoyable transportation choices among children and adolescents.

Several studies have reported parental support as a barrier. In northern Sweden, most adolescents reported receiving considerable support from their parents, school and peers for walking and cycling to school. Parents play a crucial role in promoting AST among adolescents. Therefore, efforts should be made to encourage parental support for walking, cycling, and riding non-motorised scooters to school. However, according to participants' views, most parents and peers do not support using a non-motorised scooter. Our study shows that almost all of the participants have at least one bicycle at home, which suggests parental support for cycling. This is considered a significant enabler for them to cycle to school. However, only about half of the participants reported owning a non-motorised scooter, suggesting that lack of parental support, safety concerns [29], personal norms and preferences, along with the wide availability of bicycles, might prevent them from riding non-motorised scooters to school. Another reason for low preference of non-motorised scooters could be due lack of experience of commuting to school with non-motorised scooters and the unsuitability of small scooter tires for snowy winter conditions. Hence, interventions that aim to provide training for riding non-motorised scooters may aid in decreasing these safety concerns.

School support can also play a significant role in promoting AST among adolescents. Therefore, efforts should be made to encourage school support for walking, cycling, and riding non-motorised scooters to school. For instance, providing cycling and walking incentives may encourage adolescents to use active transport more frequently. Therefore, schools can participate in interventions incorporating game design elements such as competition, badges, leaderboards, and rewards to encourage AST among adolescents. However, more research is needed to develop effective interventions that can promote AST in different contexts.

Safety concerns have been reported to be a barrier for AST in many countries [6,9]. However, in northern Sweden, most parents and adolescents feel it is safe to use AST. Although most adolescents in northern Sweden feel safe using AST, it is still important to address safety concerns of the few associated with walking, cycling, and riding non-motorised scooters to school. Interventions such as providing winter tires for bicycles and clearing snow in time can help to reduce safety concerns during winter. Also, although many adolescents in the study are acknowledging the presence of foot paths and bicycle racks in schools and public places, creating more bike lanes and pedestrian-friendly infrastructure could reduce perceived safety concerns among adolescents, making it easier for them to walk or cycle to school.

Consistent with previous studies, cold and wet conditions were seen as a barrier to AST [1,25,30]. Many adolescents indicated that the cold and wet weather makes it hard to walk, cycle, or ride a non-motorised vehicle to school. Consequently, more participants used motorised transport during the winter season than during the summer. Therefore, cleaning snow in time and equipping bicycles with winter tires might motivate more adolescents to use AST during winter. In addition, most adolescents own waterproof raincoats, which can help them feel motivated to use AST even on wet days. Moreover, previous research has shown that involvement and togetherness can be powerful motivators toward the use of AST in winter conditions [1].

Personal barriers, such as lack of motivation needs to be addressed to promote AST among adolescents. For instance, efforts could be made to make walking, cycling, and riding non-motorised scooters to school more fun and engaging. Many children like the idea of having fun when going to school [6,31]. One way of making AST fun is by using games and gamification to motivate more kids to use AST [4,10,[31], [32], [33]]. Several studies that used games and gamification on children to promote AST received positive feedback from parents, peers, and school officials and noted that gamification helped children feel motivated and engaged using AST [10,34]. There are various motivations behind people's participation in gamification-based interventions such as competition, gaining rewards, socialising, and exploring their local area [30]. Despite the number of game design techniques used in practice, the concept of gamification needs to be better established, and there is a need for more theoretical frameworks in developing these interventions in the context of AST [35].

We acknowledge the following limitations of this study: The data was not collected from parents and teachers, so some results regarding parents and school are merely suggestive. Furthermore, the questionnaire translation was done by one researcher, a native Swedish speaker who is fluent in English, and no other person validated the translation. There was no validation of the data about the distance to school, the data collected were based on the estimates by the adolescents. Moreover, the study used a sample collected in a single school in a small city in northern Sweden, thus limiting its findings’ scope. Consequently, the results cannot be used to generalise the AST situations in northern Sweden. Therefore, we believe that more studies are needed to analyse the issue of AST in Sweden and other countries.

## 5 Conclusion

This study aimed to analyse the factors that influence the choice of AST among adolescents in northern Sweden. As a result, we identified multiple factors influencing adolescents’ motivation towards AST through the collected data. For example, walking and cycling to school are more common among adolescents and are considered a great way to exercise than riding a non-motorised scooter to school. In addition, most adolescents are more confident about walking and cycling to school. However, the findings also show there is minimal support from parents, peers, and schools for riding non-motorised scooters compared to walking and cycling to school. Furthermore, many adolescents avoid using AST due to time constraints, winter conditions and distance to school. Interventions are needed to encourage AST use, especially during the winter season. Using gamification to motivate adolescents to use AST is a promising method towards this objective, especially if the program involves parents and schools in its implementation.

The findings could inform the development of interventions aimed at motivating and promoting the use of AST in children, particularly adolescents. The study shows it is essential to have interventions that spark children's motivation and enthusiasm to use AST. These should include developing a positive and fun AST culture and creating safe and supportive environments. The findings of this study will guide us in designing a persuasive gamification system that will motivate and engage adolescents into using AST. The study could also guide researchers and policymakers in designing interventions that would improve the use of AST.

## Funding statement

This study was partly supported by Vinnova (grant 2020 - 01867) and by the International Cooperation Joint Research Fund of 10.13039/501100002619Ajou University (S-2023-G0001-00020).

## Data availability statement

The data associated with this study has not been deposited into a publicly available repository. However, data supporting the findings of this study will be made available upon request. Please contact first or second author for inquiries regarding the data.

## Ethics declarations

This study was conducted in accordance with the ethical standards outlined in the Declaration of Helsinki. This study was reviewed and approved by regional ethical committee in Umeå, with the approval number: DR 2018-10-31 M. All participants legal guardians provided informed consent to participate in the study. This research did not involve any experimentation on animals. No animal subjects were used for any aspect of this study. The authors of this article have made significant contributions to the research design, data collection, analysis, and manuscript preparation, adhering to recognized ethical guidelines for academic authorship. The authors declare no conflicts of interest that could influence the research, data interpretation, or presentation of results.

## CRediT authorship contribution statement

**Nuru Jingili:** Data curation, Formal analysis, Methodology, Visualization, Writing – original draft, Writing – review & editing. **Solomon Sunday Oyelere:** Conceptualization, Investigation, Methodology, Software, Supervision, Validation, Writing – review & editing. **Simon Malmström Berghem:** Data curation, Formal analysis, Investigation, Resources, Software, Visualization. **Robert Brännström:** Conceptualization, Funding acquisition, Investigation, Project administration, Resources, Software, Supervision. **Teemu H. Laine:** Conceptualization, Funding acquisition, Investigation, Resources, Supervision, Visualization, Writing – review & editing. **Oluwafemi Samson Balogun:** Data curation, Investigation, Methodology, Software, Validation, Visualization, Writing – review & editing.

## Declaration of competing interest

The authors declare that they have no known competing financial interests or personal relationships that could have appeared to influence the work reported in this paper.

## Figures and Tables

**Appendix 1:** Multiple Comparison table using Duncan test for Table 8

Multiple Comparisons							
LSD							
Dependent Variable	(I) Group	(J) Group	Mean Difference (I-J)	Std. Error	Sig.	95% Confidence Interval	
						Lower Bound	Upper Bound
Interesting	Walking	Cycling	.98571^∗^	.22958	.000	.5331	1.4383
		NonMotorisedScooter	-.55714^∗^	.22958	.016	-1.0098	-.1045
	Cycling	Walking	-.98571^∗^	.22958	.000	-1.4383	-.5331
		NonMotorisedScooter	-1.54286^∗^	.22958	.000	-1.9955	-1.0902
	NonMotorisedScooter	Walking	.55714^∗^	.22958	.016	.1045	1.0098
		Cycling	1.54286^∗^	.22958	.000	1.0902	1.9955
Pleasant	Walking	Cycling	-.21429^∗^	.07532	.005	-.3628	-.0658
		NonMotorisedScooter	.17143^∗^	.07532	.024	.0229	.3199
	Cycling	Walking	.21429^∗^	.07532	.005	.0658	.3628
		NonMotorisedScooter	.38571^∗^	.07532	.000	.2372	.5342
	NonMotorisedScooter	Walking	-.17143^∗^	.07532	.024	-.3199	-.0229
		Cycling	-.38571^∗^	.07532	.000	-.5342	-.2372
Boring	Walking	Cycling	-.22857^∗^	.07846	.004	-.3833	-.0739
		NonMotorisedScooter	.24286^∗^	.07846	.002	.0882	.3975
	Cycling	Walking	.22857^∗^	.07846	.004	.0739	.3833
		NonMotorisedScooter	.47143^∗^	.07846	.000	.3167	.6261
	NonMotorisedScooter	Walking	-.24286^∗^	.07846	.002	-.3975	-.0882
		Cycling	-.47143^∗^	.07846	.000	-.6261	-.3167
Health	Walking	Cycling	-.02857	.05245	.586	-.1320	.0748
		NonMotorisedScooter	.31429^∗^	.05245	.000	.2109	.4177
	Cycling	Walking	.02857	.05245	.586	-.0748	.1320
		NonMotorisedScooter	.34286^∗^	.05245	.000	.2395	.4463
	NonMotorisedScooter	Walking	-.31429^∗^	.05245	.000	-.4177	-.2109
		Cycling	-.34286^∗^	.05245	.000	-.4463	-.2395
Good	Walking	Cycling	-.12857^∗^	.06462	.048	-.2560	-.0012
		NonMotorisedScooter	.35714^∗^	.06462	.000	.2297	.4845
	Cycling	Walking	.12857^∗^	.06462	.048	.0012	.2560
		NonMotorisedScooter	.48571^∗^	.06462	.000	.3583	.6131
	NonMotorisedScooter	Walking	-.35714^∗^	.06462	.000	-.4845	-.2297
		Cycling	-.48571^∗^	.06462	.000	-.6131	-.3583
Use	Walking	Cycling	.12857	.18197	.481	-.2302	.4873
		NonMotorisedScooter	-.85714^∗^	.18197	.000	-1.2159	-.4984
	Cycling	Walking	-.12857	.18197	.481	-.4873	.2302
		NonMotorisedScooter	-.98571^∗^	.18197	.000	-1.3445	-.6270
	NonMotorisedScooter	Walking	.85714^∗^	.18197	.000	.4984	1.2159
		Cycling	.98571^∗^	.18197	.000	.6270	1.3445
MyparentGuardian	Walking	Cycling	-.60000	.31696	.060	-1.2249	.0249
		NonMotorisedScooter	1.95714^∗^	.31696	.000	1.3323	2.5820
	Cycling	Walking	.60000	.31696	.060	-.0249	1.2249
		NonMotorisedScooter	2.55714^∗^	.31696	.000	1.9323	3.1820
	NonMotorisedScooter	Walking	-1.95714^∗^	.31696	.000	-2.5820	-1.3323
		Cycling	-2.55714^∗^	.31696	.000	-3.1820	-1.9323
Myfriend	Walking	Cycling	-.62857^∗^	.29826	.036	-1.2166	-.0406
		NonMotorisedScooter	1.45714^∗^	.29826	.000	.8691	2.0452
	Cycling	Walking	.62857^∗^	.29826	.036	.0406	1.2166
		NonMotorisedScooter	2.08571^∗^	.29826	.000	1.4977	2.6737
	NonMotorisedScooter	Walking	-1.45714^∗^	.29826	.000	-2.0452	-.8691
		Cycling	-2.08571^∗^	.29826	.000	-2.6737	-1.4977
Oneboth	Walking	Cycling	-.21429	.33140	.519	-.8676	.4391
		NonMotorisedScooter	2.70000^∗^	.33140	.000	2.0466	3.3534
	Cycling	Walking	.21429	.33140	.519	-.4391	.8676
		NonMotorisedScooter	2.91429^∗^	.33140	.000	2.2609	3.5676
	NonMotorisedScooter	Walking	-2.70000^∗^	.33140	.000	-3.3534	-2.0466
		Cycling	-2.91429^∗^	.33140	.000	-3.5676	-2.2609
Itisconsidered	Walking	Cycling	-.04286	.31003	.890	-.6541	.5684
		NonMotorisedScooter	-.34286	.31003	.270	-.9541	.2684
	Cycling	Walking	.04286	.31003	.890	-.5684	.6541
		NonMotorisedScooter	-.30000	.31003	.334	-.9112	.3112
	NonMotorisedScooter	Walking	.34286	.31003	.270	-.2684	.9541
		Cycling	.30000	.31003	.334	-.3112	.9112
Nootherstudent	Walking	Cycling	.01429	.32859	.965	-.6335	.6621
		NonMotorisedScooter	-1.85714^∗^	.32859	.000	-2.5050	-1.2093
	Cycling	Walking	-.01429	.32859	.965	-.6621	.6335
		NonMotorisedScooter	-1.87143^∗^	.32859	.000	-2.5192	-1.2236
	NonMotorisedScooter	Walking	1.85714^∗^	.32859	.000	1.2093	2.5050
		Cycling	1.87143^∗^	.32859	.000	1.2236	2.5192
Myschool	Walking	Cycling	-.11429	.29887	.703	-.7035	.4749
		NonMotorisedScooter	1.11429^∗^	.29887	.000	.5251	1.7035
	Cycling	Walking	.11429	.29887	.703	-.4749	.7035
		NonMotorisedScooter	1.22857^∗^	.29887	.000	.6394	1.8178
	NonMotorisedScooter	Walking	-1.11429^∗^	.29887	.000	-1.7035	-.5251
		Cycling	-1.22857^∗^	.29887	.000	-1.8178	-.6394
Iamconfident	Walking	Cycling	-1.41429^∗^	.35010	.000	-2.1045	-.7241
		NonMotorisedScooter	.10000	.35010	.775	-.5902	.7902
	Cycling	Walking	1.41429^∗^	.35010	.000	.7241	2.1045
		NonMotorisedScooter	1.51429^∗^	.35010	.000	.8241	2.2045
	NonMotorisedScooter	Walking	-.10000	.35010	.775	-.7902	.5902
		Cycling	-1.51429^∗^	.35010	.000	-2.2045	-.8241
Ihavecompletecontrol	Walking	Cycling	-.22857	.33587	.497	-.8907	.4336
		NonMotorisedScooter	1.14286^∗^	.33587	.001	.4807	1.8050
	Cycling	Walking	.22857	.33587	.497	-.4336	.8907
		NonMotorisedScooter	1.37143^∗^	.33587	.000	.7093	2.0336
	NonMotorisedScooter	Walking	-1.14286^∗^	.33587	.001	-1.8050	-.4807
		Cycling	-1.37143^∗^	.33587	.000	-2.0336	-.7093
Ioften	Walking	Cycling	-.95714^∗^	.34049	.005	-1.6284	-.2859
		NonMotorisedScooter	2.10000^∗^	.34049	.000	1.4287	2.7713
	Cycling	Walking	.95714^∗^	.34049	.005	.2859	1.6284
		NonMotorisedScooter	3.05714^∗^	.34049	.000	2.3859	3.7284
	NonMotorisedScooter	Walking	-2.10000^∗^	.34049	.000	-2.7713	-1.4287
		Cycling	-3.05714^∗^	.34049	.000	-3.7284	-2.3859
Amthinking	Walking	Cycling	-.68571^∗^	.33333	.041	-1.3429	-.0286
		NonMotorisedScooter	2.44286^∗^	.33333	.000	1.7857	3.1000
	Cycling	Walking	.68571^∗^	.33333	.041	.0286	1.3429
		NonMotorisedScooter	3.12857^∗^	.33333	.000	2.4714	3.7857
	NonMotorisedScooter	Walking	-2.44286^∗^	.33333	.000	-3.1000	-1.7857
		Cycling	-3.12857^∗^	.33333	.000	-3.7857	-2.4714

*. The mean difference is significant at the 0.05 level.

**Appendix 2:** Multiple Comparison table using Duncan test for Table 9

Multiple Comparisons							
LSD							
Dependent Variable	(I) Group	(J) Group	Mean Difference (I-J)	Std. Error	Sig.	95% Confidence Interval	
						Lower Bound	Upper Bound
Exercise	Walking	Cycling	-.06154	.28966	.832	-.6329	.5098
		NonMotorisedScooter	1.89231^∗^	.28966	.000	1.3210	2.4636
	Cycling	Walking	.06154	.28966	.832	-.5098	.6329
		NonMotorisedScooter	1.95385^∗^	.28966	.000	1.3825	2.5252
	NonMotorisedScooter	Walking	-1.89231^∗^	.28966	.000	-2.4636	-1.3210
		Cycling	-1.95385^∗^	.28966	.000	-2.5252	-1.3825
TooMuchTime	Walking	Cycling	1.92308^∗^	.41878	.000	1.0971	2.7491
		NonMotorisedScooter	.89231^∗^	.41878	.034	.0663	1.7183
	Cycling	Walking	-1.92308^∗^	.41878	.000	-2.7491	-1.0971
		NonMotorisedScooter	-1.03077^∗^	.41878	.015	-1.8568	-.2048
	NonMotorisedScooter	Walking	-.89231^∗^	.41878	.034	-1.7183	-.0663
		Cycling	1.03077^∗^	.41878	.015	.2048	1.8568
TooMuchPlanning	Walking	Cycling	.73846	.37911	.053	-.0093	1.4862
		NonMotorisedScooter	.23077	.37911	.543	-.5170	.9785
	Cycling	Walking	-.73846	.37911	.053	-1.4862	.0093
		NonMotorisedScooter	-.50769	.37911	.182	-1.2554	.2401
	NonMotorisedScooter	Walking	-.23077	.37911	.543	-.9785	.5170
		Cycling	.50769	.37911	.182	-.2401	1.2554
HotAndSweaty	Walking	Cycling	-.43077	.36922	.245	-1.1590	.2975
		NonMotorisedScooter	.00000	.36922	1.000	-.7283	.7283
	Cycling	Walking	.43077	.36922	.245	-.2975	1.1590
		NonMotorisedScooter	.43077	.36922	.245	-.2975	1.1590
	NonMotorisedScooter	Walking	.00000	.36922	1.000	-.7283	.7283
		Cycling	-.43077	.36922	.245	-1.1590	.2975
StuffToCarry	Walking	Cycling	.47692	.38955	.222	-.2914	1.2453
		NonMotorisedScooter	-.15385	.38955	.693	-.9222	.6145
	Cycling	Walking	-.47692	.38955	.222	-1.2453	.2914
		NonMotorisedScooter	-.63077	.38955	.107	-1.3991	.1376
	NonMotorisedScooter	Walking	.15385	.38955	.693	-.6145	.9222
		Cycling	.63077	.38955	.107	-.1376	1.3991
NotConvenientAfterSchool	Walking	Cycling	.32308	.35110	.359	-.3694	1.0156
		NonMotorisedScooter	.13846	.35110	.694	-.5541	.8310
	Cycling	Walking	-.32308	.35110	.359	-1.0156	.3694
		NonMotorisedScooter	-.18462	.35110	.600	-.8771	.5079
	NonMotorisedScooter	Walking	-.13846	.35110	.694	-.8310	.5541
		Cycling	.18462	.35110	.600	-.5079	.8771
Tired	Walking	Cycling	.44615	.39975	.266	-.3423	1.2346
		NonMotorisedScooter	.18462	.39975	.645	-.6038	.9731
	Cycling	Walking	-.44615	.39975	.266	-1.2346	.3423
		NonMotorisedScooter	-.26154	.39975	.514	-1.0500	.5269
	NonMotorisedScooter	Walking	-.18462	.39975	.645	-.9731	.6038
		Cycling	.26154	.39975	.514	-.5269	1.0500
CantBother	Walking	Cycling	.78462	.42217	.065	-.0481	1.6173
		NonMotorisedScooter	-.53846	.42217	.204	-1.3711	.2942
	Cycling	Walking	-.78462	.42217	.065	-1.6173	.0481
		NonMotorisedScooter	-1.32308^∗^	.42217	.002	-2.1558	-.4904
	NonMotorisedScooter	Walking	.53846	.42217	.204	-.2942	1.3711
		Cycling	1.32308^∗^	.42217	.002	.4904	2.1558
Far	Walking	Cycling	1.21538^∗^	.41207	.004	.4026	2.0281
		NonMotorisedScooter	.55385	.41207	.181	-.2589	1.3666
	Cycling	Walking	-1.21538^∗^	.41207	.004	-2.0281	-.4026
		NonMotorisedScooter	-.66154	.41207	.110	-1.4743	.1512
	NonMotorisedScooter	Walking	-.55385	.41207	.181	-1.3666	.2589
		Cycling	.66154	.41207	.110	-.1512	1.4743
NoPaths	Walking	Cycling	-.04615	.27097	.865	-.5806	.4883
		NonMotorisedScooter	-.27692	.27097	.308	-.8114	.2575
	Cycling	Walking	.04615	.27097	.865	-.4883	.5806
		NonMotorisedScooter	-.23077	.27097	.395	-.7652	.3037
	NonMotorisedScooter	Walking	.27692	.27097	.308	-.2575	.8114
		Cycling	.23077	.27097	.395	-.3037	.7652
TooColdAndWet	Walking	Cycling	-.29231	.42828	.496	-1.1371	.5524
		NonMotorisedScooter	-.52308	.42828	.223	-1.3678	.3217
	Cycling	Walking	.29231	.42828	.496	-.5524	1.1371
		NonMotorisedScooter	-.23077	.42828	.591	-1.0755	.6140
	NonMotorisedScooter	Walking	.52308	.42828	.223	-.3217	1.3678
		Cycling	.23077	.42828	.591	-.6140	1.0755
Unsafe	Walking	Cycling	.29231	.31820	.359	-.3353	.9199
		NonMotorisedScooter	-.26154	.31820	.412	-.8892	.3661
	Cycling	Walking	-.29231	.31820	.359	-.9199	.3353
		NonMotorisedScooter	-.55385	.31820	.083	-1.1815	.0738
	NonMotorisedScooter	Walking	.26154	.31820	.412	-.3661	.8892
		Cycling	.55385	.31820	.083	-.0738	1.1815
MyParentsThinkUnsafe	Walking	Cycling	.07692	.29154	.792	-.4981	.6520
		NonMotorisedScooter	-.41538	.29154	.156	-.9904	.1596
	Cycling	Walking	-.07692	.29154	.792	-.6520	.4981
		NonMotorisedScooter	-.49231	.29154	.093	-1.0673	.0827
	NonMotorisedScooter	Walking	.41538	.29154	.156	-.1596	.9904
		Cycling	.49231	.29154	.093	-.0827	1.0673

*. The mean difference is significant at the 0.05 level.
