# Supplementary material for: Adolescents’ perceptions of active school transport in northern Sweden
Source: Heliyon. 2023 Oct 7;9(10):e20779. doi: 10.1016/j.heliyon.2023.e20779 (PMC10582399; doi:10.1016/j.heliyon.2023.e20779)
Supplement: Multimedia component 1 [file mmc1.docx]

Title: Adolescents’ perceptions of active school transport in Northern Sweden

**Questionnaire**

| **Sociodemographic characteristics** | **Options/Likert-scale** |
| --- | --- |
| Age  Gender  Estimated distance to school (km)  Number of bicycles at home  Number of vehicles at home  Number of motorised scooters at home  Number of non-motorised scooters at home  Number of waterproof raincoat at home |  |
| **Travel to school habit** |  |
| How do you usually travel to school during summer?  By car (driven by others)  By car (driven by myself)  By school bus  By public transport  On foot  By bike  By motorized scooter  By non-motorized scooter  By skateboard  Other, please specify | “never”, “rarely”, “sometimes”, “most of the time” and “all of the time” |
| How do you usually travel to school during winter?  By car (driven by others)  By car (driven by myself)  By school bus  By public transport  On foot  By bike  By motorized scooter  By non-motorized scooter  By skateboard  Other, please specify | “never”, “rarely”, “sometimes”, “most of the time” and “all of the time” |
| How often do you walk to school in the previous two weeks? | “never”, “almost never”, “sometimes”, “almost everyday” or “everyday” |
| How often do you cycle to school in the previous two weeks? | “never”, “almost never”, “sometimes”, “almost everyday” or “everyday” |
| How often do you ride a non-motorized scooter to school in the previous two weeks? | “never”, “almost never”, “sometimes”, “almost everyday” or “everyday” |
| Estimate how long would your journey to school take if you walk to school | “1–5 min”, “6–10min”, 11–20 min”, “21–30 min”, “31þ _min” _or “I don’t know”). |
| Estimate how long would your journey to school take if you cycle to school | “1–5 min”, “6–10min”, 11–20 min”, “21–30 min”, “31þ _min” _or “I don’t know”). |
| Estimate how long would your journey to school take if you ride a non-motorized scooter to school | “1–5 min”, “6–10min”, 11–20 min”, “21–30 min”, “31þ _min” _or “I don’t know”). |
| **Attitudes towards walking, cycling and riding non-motorized to school** |  |
| For me, regularly walking to school would be interesting | ”strongly disagree, disagree, somewhat disagree, somewhat agree, agree, strongly agree” |
| For me, regularly cycling to school would be interesting | ”strongly disagree, disagree, somewhat disagree, somewhat agree, agree, strongly agree” |
| For me, regularly riding a non-motorised scooter to school would be interesting | ”strongly disagree, disagree, somewhat disagree, somewhat agree, agree, strongly agree” |
| For me, regularly walking to school would be pleasant | ”strongly disagree, disagree, somewhat disagree, somewhat agree, agree, strongly agree” |
| For me, regularly cycling to school would be pleasant | ”strongly disagree, disagree, somewhat disagree, somewhat agree, agree, strongly agree” |
| For me, regularly riding non-motorised scooter to school would be pleasant | ”strongly disagree, disagree, somewhat disagree, somewhat agree, agree, strongly agree” |
| For me, regularly walking to school would be boring | ”strongly disagree, disagree, somewhat disagree, somewhat agree, agree, strongly agree” |
| For me, regularly cycling to school would be boring | ”strongly disagree, disagree, somewhat disagree, somewhat agree, agree, strongly agree” |
| For me, regularly riding non-motorised scooter to school would be boring | ”strongly disagree, disagree, somewhat disagree, somewhat agree, agree, strongly agree” |
| For me, regularly walking to school would be healthy | ”strongly disagree, disagree, somewhat disagree, somewhat agree, agree, strongly agree” |
| For me, regularly cycling to school would be healthy | ”strongly disagree, disagree, somewhat disagree, somewhat agree, agree, strongly agree” |
| For me, regularly riding non-motorised scooter to school would be healthy | ”strongly disagree, disagree, somewhat disagree, somewhat agree, agree, strongly agree” |
| For me, regularly walking to school would be good | ”strongly disagree, disagree, somewhat disagree, somewhat agree, agree, strongly agree” |
| For me, regularly cycling to school would be good | ”strongly disagree, disagree, somewhat disagree, somewhat agree, agree, strongly agree” |
| For me, regularly riding non-motorised scooter to school would be good | ”strongly disagree, disagree, somewhat disagree, somewhat agree, agree, strongly agree” |
| For me, regularly walking to school would be useful | ”strongly disagree, disagree, somewhat disagree, somewhat agree, agree, strongly agree” |
| For me, regularly cycling to school would be useful | ”strongly disagree, disagree, somewhat disagree, somewhat agree, agree, strongly agree” |
| For me, regularly riding a non-motorised scooter to school would be useful | ”strongly disagree, disagree, somewhat disagree, somewhat agree, agree, strongly agree” |
| **Subjective/perceived norm** |  |
| My parents or guardians think I should walk to school | ”strongly disagree, disagree, somewhat disagree, neutral, somewhat agree, agree, strongly agree” |
| My parents or guardians think I should ride a bicycle to school | ”strongly disagree, disagree, somewhat disagree, neutral, somewhat agree, agree, strongly agree” |
| My parents or guardians think I should ride non-motorized scooter to school | ” ”strongly disagree, disagree, somewhat disagree, neutral, somewhat agree, agree, strongly agree” |
| My friends think I think I should walk to school | ”strongly disagree, disagree, somewhat disagree, neutral, somewhat agree, agree, strongly agree” |
| My friends think I should ride a bicycle to school | ”strongly disagree, disagree, somewhat disagree, neutral, somewhat agree, agree, strongly agree” |
| My friends think I should ride non-motorized scooter to school | ”strongly disagree, disagree, somewhat disagree, neutral, somewhat agree, agree, strongly agree” |
| One or both of my parents or guardians walk frequently | ”strongly disagree, disagree, somewhat disagree, neutral, somewhat agree, agree, strongly agree” |
| One or both of my parents or guardians ride a bicycle frequently | ”strongly disagree, disagree, somewhat disagree, neutral, somewhat agree, agree, strongly agree” |
| One or both of my parents or guardians ride a non-motorized scooter frequently | ”strongly disagree, disagree, somewhat disagree, neutral, somewhat agree, agree, strongly agree” |
| It is not considered cool to walk to school | ”strongly disagree, disagree, somewhat disagree, neutral, somewhat agree, agree, strongly agree” |
| It is not considered cool to ride a bicycle to school | ”strongly disagree, disagree, somewhat disagree, neutral, somewhat agree, agree, strongly agree” |
| It is not considered cool to ride a non-motorized bicycle to school | ”strongly disagree, disagree, somewhat disagree, neutral, somewhat agree, agree, strongly agree” |
| No other students walk to school | ”strongly disagree, disagree, somewhat disagree, neutral, somewhat agree, agree, strongly agree” |
| No other students ride a bicycle to school | ”strongly disagree, disagree, somewhat disagree, neutral, somewhat agree, agree, strongly agree” |
| No other students ride a non-motorized scooter to school | ”strongly disagree, disagree, somewhat disagree, neutral, somewhat agree, agree, strongly agree” |
| Number of friends who always or sometimes walk to school | “0 to 5” |
| Number of friends who always or sometimes ride a bicycle to school | “0 to 5” |
| Number of friends who always or sometimes ride a non-motorized scooter to school | “0 to 5” |
| My school encourages me to walk to school | ”strongly disagree, disagree, somewhat disagree, neutral, somewhat agree, agree, strongly agree” |
| My school encourages me to ride a bicycle to school | ”strongly disagree, disagree, somewhat disagree, neutral, somewhat agree, agree, strongly agree” |
| My school encourages me to ride a non-motorized scooter to school | ”strongly disagree, disagree, somewhat disagree, neutral, somewhat agree, agree, strongly agree” |
| I am confident I could walk to school | ”strongly disagree, disagree, somewhat disagree, neutral, somewhat agree, agree, strongly agree” |
| I am confident I could ride a bicycle to school | ”strongly disagree, disagree, somewhat disagree, neutral, somewhat agree, agree, strongly agree” |
| I am confident I could ride a scooter to school | ”strongly disagree, disagree, somewhat disagree, neutral, somewhat agree, agree, strongly agree” |
| I have complete control over whether or not I walk to school | ”strongly disagree, disagree, somewhat disagree, neutral, somewhat agree, agree, strongly agree” |
| I have complete control over whether or not I ride a bicycle to school | ”strongly disagree, disagree, somewhat disagree, neutral, somewhat agree, agree, strongly agree” |
| I have complete control over whether or not I ride a non-motorized scooter to school | ”strongly disagree, disagree, somewhat disagree, neutral, somewhat agree, agree, strongly agree” |
| I want to regularly walk to school | ”strongly disagree, disagree, somewhat disagree, neutral, somewhat agree, agree, strongly agree” |
| I want to regularly cycle to school | ”strongly disagree, disagree, somewhat disagree, neutral, somewhat agree, agree, strongly agree” |
| I want to regularly ride a non-motorized scooter to school | ”strongly disagree, disagree, somewhat disagree, neutral, somewhat agree, agree, strongly agree” |
| I intend to walk to school frequently | ”strongly disagree, disagree, somewhat disagree, neutral, somewhat agree, agree, strongly agree” |
| I intend to cycle to school frequently | ”strongly disagree, disagree, somewhat disagree, neutral, somewhat agree, agree, strongly agree” |
| I intend to ride a non-motorized scooter to school frequently | ”strongly disagree, disagree, somewhat disagree, neutral, somewhat agree, agree, strongly agree” |
| **POST questionnaire starts here** |  |
| **Personal incentives** |  |
| Walking to school is a great way to get some exercise | ”strongly disagree, disagree, somewhat disagree, neutral, somewhat agree, agree, strongly agree” |
| Riding a bicycle to school is a great way to get some exercise | ”strongly disagree, disagree, somewhat disagree, neutral, somewhat agree, agree, strongly agree” |
| Riding a non-motorized scooter is a great way to get some exercise | ”strongly disagree, disagree, somewhat disagree, neutral, somewhat agree, agree, strongly agree” |
| **Personal barriers** |  |
| Walking to school takes too much time | ”strongly disagree, disagree, somewhat disagree, neutral, somewhat agree, agree, strongly agree” |
| Cycling to school takes too much time | ”strongly disagree, disagree, somewhat disagree, neutral, somewhat agree, agree, strongly agree” |
| Riding a non-motorized scooter to school takes too much time | ”strongly disagree, disagree, somewhat disagree, neutral, somewhat agree, agree, strongly agree” |
| It involves too much planning ahead to walk to school | ”strongly disagree, disagree, somewhat disagree, neutral, somewhat agree, agree, strongly agree” |
| It involves too much planning ahead to cycle to school | ”strongly disagree, disagree, somewhat disagree, neutral, somewhat agree, agree, strongly agree” |
| It involves too much planning ahead to ride a non-motorized scooter to school | ”strongly disagree, disagree, somewhat disagree, neutral, somewhat agree, agree, strongly agree” |
| I get too hot and sweaty walking to school | ”strongly disagree, disagree, somewhat disagree, neutral, somewhat agree, agree, strongly agree” |
| I get too hot and sweaty cycling to school | ”strongly disagree, disagree, somewhat disagree, neutral, somewhat agree, agree, strongly agree” |
| I get too hot and sweaty riding a non-motorized scooter to school | ”strongly disagree, disagree, somewhat disagree, neutral, somewhat agree, agree, strongly agree” |
| I have too much stuff to carry to walk to school | ”strongly disagree, disagree, somewhat disagree, neutral, somewhat agree, agree, strongly agree” |
| I have too much stuff to carry to cycle to school | ”strongly disagree, disagree, somewhat disagree, neutral, somewhat agree, agree, strongly agree” |
| I have too much stuff to carry to ride a non-motorized scooter to school | ”strongly disagree, disagree, somewhat disagree, neutral, somewhat agree, agree, strongly agree” |
| It is not convenient for me to walk to school because of my after-school schedule | ”strongly disagree, disagree, somewhat disagree, neutral, somewhat agree, agree, strongly agree” |
| It is not convenient for me to cycle to school because of my after-school schedule | ”strongly disagree, disagree, somewhat disagree, neutral, somewhat agree, agree, strongly agree” |
| It is not convenient for me to ride a non-motorized scooter to school because of my after-school schedule | ”strongly disagree, disagree, somewhat disagree, neutral, somewhat agree, agree, strongly agree” |
| I often feel too tired to walk to school | ”strongly disagree, disagree, somewhat disagree, neutral, somewhat agree, agree, strongly agree” |
| I often feel too tired to cycle to school | ”strongly disagree, disagree, somewhat disagree, neutral, somewhat agree, agree, strongly agree” |
| I often feel too tired to ride a non-motorized scooter to school | ”strongly disagree, disagree, somewhat disagree, neutral, somewhat agree, agree, strongly agree” |
| I often cannot be bothered to walk to school | ”strongly disagree, disagree, somewhat disagree, neutral, somewhat agree, agree, strongly agree” |
| I often cannot be bothered to cycle to school | ”strongly disagree, disagree, somewhat disagree, neutral, somewhat agree, agree, strongly agree” |
| I often cannot be bothered to ride a non-motorized scooter to school | ”strongly disagree, disagree, somewhat disagree, neutral, somewhat agree, agree, strongly agree” |
| **Environmental factors** |  |
| It is too far to walk to school | ”strongly disagree, disagree, somewhat disagree, neutral, somewhat agree, agree, strongly agree” |
| It is too far to cycle to school | ”strongly disagree, disagree, somewhat disagree, neutral, somewhat agree, agree, strongly agree” |
| It is too far to ride a non-motorized scooter to school | ”strongly disagree, disagree, somewhat disagree, neutral, somewhat agree, agree, strongly agree” |
| There are no footpaths along the way | ”strongly disagree, disagree, somewhat disagree, neutral, somewhat agree, agree, strongly agree” |
| There are no cycle paths along the way | ”strongly disagree, disagree, somewhat disagree, neutral, somewhat agree, agree, strongly agree” |
| There are no non-motorized scooter paths along the way | ”strongly disagree, disagree, somewhat disagree, neutral, somewhat agree, agree, strongly agree” |
| The weather is too cold and wet to walk | ”strongly disagree, disagree, somewhat disagree, neutral, somewhat agree, agree, strongly agree” |
| The weather is too cold and wet to cycle | ”strongly disagree, disagree, somewhat disagree, neutral, somewhat agree, agree, strongly agree” |
| The weather is too cold and wet to ride non-motorized scooter | ”strongly disagree, disagree, somewhat disagree, neutral, somewhat agree, agree, strongly agree” |
| **Safety perceptions** |  |
| It is unsafe to walk to school | ”strongly disagree, disagree, somewhat disagree, neutral, somewhat agree, agree, strongly agree” |
| It is unsafe to cycle to school | ”strongly disagree, disagree, somewhat disagree, neutral, somewhat agree, agree, strongly agree” |
| It is unsafe to ride non-motorized scooter to school | ”strongly disagree, disagree, somewhat disagree, neutral, somewhat agree, agree, strongly agree” |
| My parents think it is not safe to walk to school | ”strongly disagree, disagree, somewhat disagree, neutral, somewhat agree, agree, strongly agree” |
| My parents think it is not safe to cycle to school | ”strongly disagree, disagree, somewhat disagree, neutral, somewhat agree, agree, strongly agree” |
| My parents think it is not safe to ride non-motorized scooter to school | ”strongly disagree, disagree, somewhat disagree, neutral, somewhat agree, agree, strongly agree” |
